# First Vasectomy Procedure Successfully Performed on a Southern African Male Giraffe (*Giraffa camelopardalis giraffa*)

**DOI:** 10.1002/zoo.70046

**Published:** 2025-12-16

**Authors:** Francois Deacon, Willem Daffue, Edward Collin Albertyn, Susanna Maria Krüger, Ayanda Maqhashu, Jacquiline Goedhals, Andri Grobbelaar, Marike Badenhorst

**Affiliations:** ^1^ Department of Animal Science, Faculty of Natural and Agricultural Sciences University of the Free State Bloemfontein South Africa; ^2^ Kroonstad Animal Hospital Kroonstad South Africa; ^3^ Absolute Genetics Bloemfontein South Africa; ^4^ Pathcare Bloemfontein South Africa; ^5^ Department of AnatomicalPathology, Faculty of Health Sciences University of the Free State Bloemfontein South Africa

**Keywords:** histopathological examinations, post‐vasectomy, reproductive health, reproductive studies, surgery, teaser male, vasectomy, wildlife contraception

## Abstract

This study reports the first successful vasectomy on a southern African male giraffe (*Giraffa camelopardalis giraffa*). The goal of the procedure was to create a teaser male for reproductive studies. Following chemical immobilization using a combination of medetomidine and thiafentanil, the giraffe underwent surgery with local anesthesia. The vas deferens were bilaterally located, dissected, and transected. Histopathological examination confirmed the presence of the vas deferens tissue. Five weeks post‐vasectomy, the giraffe was immobilized again for semen collection, confirming infertility. The giraffe was then used to confirm oestrus in synchronized females. Pre‐ and post‐vasectomy testosterone levels validated the procedure's effectiveness, confirming no change in the giraffe's normal behavior or attractiveness to females. The outcome was successful, with the giraffe rendered infertile post‐vasectomy.

## Summary

- Vasectomy in Southern African giraffes effectively rendered males infertile while maintaining normal testosterone levels and sexual behavior.
- The procedure enhances reproductive management by facilitating accurate oestrus detection in females.
- Findings demonstrate the safety and potential of vasectomy as a conservation tool for managing breeding in giraffe populations.
- This study provides a foundation for integrating vasectomy into wildlife conservation, balancing population control and breeding success.

## Graphical abstract

First successful vasectomy in a southern African male giraffe, *Giraffa camelopardis giraffa*

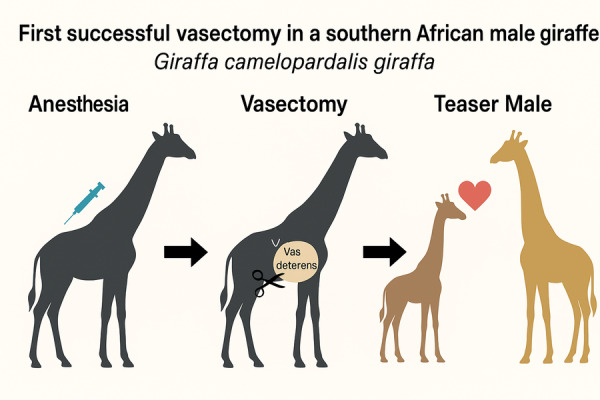

## 1 Introduction

The slow reproductive rate of giraffes (*Giraffa camelopardalis giraffa*), compounded by their long gestation period of approximately 15 months and high calf mortality, limits population growth (Deacon et al. 2015; Mitchell et al. 2009). Factors such as habitat destruction and loss of natural ecosystems are the main drivers for the decline in giraffe numbers (Bercovitch et al. 2017; Muneza et al. 2024). With some populations declining over 90% in the last three decades, results are leading to limited gene flow between subpopulations and an increase in inbreeding risks (Muller et al. 2018). To enhance reproductive performance and ensure the survival of the species, it is crucial to explore advanced reproductive techniques (Bolton et al. 2022; Comizzoli 2021; Mastromonaco and Songsasen 2020). In this context, the use of vasectomized males as teaser animals for oestrus detection and breeding enhancement is a novel approach, drawing parallels from similar practices in other species (Gill 1995; Grissett 2021; Morgan and Dawson 2008).

Teaser animals, typically sexually mature and young virgin males, are widely used in cattle, sheep, and goats to detect oestrus, a method yet to be explored in giraffes (Nowshari et al. 2005). The vasectomy procedure ensures that the male retains normal sexual behavior while being rendered infertile, making him ideal for oestrus detection without the risk of fertilization (Hess et al. 2024). In other species, vasectomy has shown no adverse effects on mating behavior, raising the question of whether the same holds for giraffes. The reversibility of vasectomies has also been proven to be possible in domesticated and wild animals (Gazendam et al. 2023; Lohiya and Tiwari 1984).

The primary aim of this pilot study was to create a vasectomized giraffe bull for effective oestrus detection in females, with a secondary aim to assess whether the procedure affects the bull's sexual behavior and testosterone levels. The goal was to develop an ideal teaser male giraffe capable of detecting oestrus without compromising its natural mating behavior, with implications for future reproductive management in giraffes. In other species, such as cattle, sheep, and goat teaser males are not only used for oestrus detection (with harnesses or heat patches), but also to induce ovulation in females due to male presence (Armstrong 2023).

## 2 Materials and Methods

### 2.1 Study Area and Focus Animal

The study was conducted during 2024 at Amanzi Private Game Reserve (28°35′50″S 26°25′46″E) in the Free State Province of South Africa. The study area consists of habitats ranging from shrublands to open grasslands, with isolated hills, and slopes (Haddad and Butler 2018). The selected focus animal was one free‐roaming, healthy, adult, male giraffe, identified as “Willem”. Based on dentition (Deacon et al. 2024; Hall‐Martin 1976), the estimated age of this animal was approximately 10–12 years and was estimated to weigh between 970 and 1400 kg (Kock et al. 2021). The study animal selection was based on fertility and the owner's request to prevent inbreeding within the herd. The male was chemically immobilized three times over a period of 5 months to assess its suitability for vasectomy (Deacon et al. 2022).

### 2.2 Immobilization Procedure for Vasectomy Candidate Selection

Following standard capture procedures, the giraffe was immobilized using (1.8 mL/18 mg) of thiafentanil (Thianil 10 mg/mL, Wildlife Pharmaceuticals, Nelspruit, South Africa) and administered intramuscularly via a Pneudart gun to propel a 2 mL Pneudart projectile syringe with a 2.5″ 13GA barbed side pointed needle, from a helicopter. Once recumbent, the giraffe was blindfolded, and its legs were secured by the experienced game capture team. The effects of Thianil were immediately reversed with 2 mL of Trexonil (Naltrexone 100 mg/mL, Wildlife Pharmaceuticals) administered intravenously (Deacon et al. 2022).

### 2.3 Semen Collection Procedure

Semen was collected using a combination of manual rectal stimulation of the accessory sex glands by hand and an El Toro Electro‐Ejaculator (El Toro 3 Electronic Ejaculator/Immobiliser for Bulls and Rams 2024), a technique involving the rectal insertion of a cattle probe of approximately 40 cm in length and 15 cm diameter to stimulate ejaculation. Each bull differs in the reaction to the settings, and in this case, the dial needed for stimulation was turned up to setting 6–10. The semen sample was evaluated by volume, color, and consistency, in addition to microscopic evaluation of mass motility, individual linear motility, and morphological parameters. Morphological evaluation was done by staining of a semen smear, whereafter abnormalities were identified and counted. By the use of Eosin‐Nigrosin stain, the number of live and dead cells was also determined. Together, these evaluations were considered to determine the fertility of males on international standard procedures for cattle and other species (Kondracki et al. 2017; Perry 2021).

Semen sample quality was evaluated based on multiple parameters, including having an ivory color (rather than gray) and a milky or creamy consistency (rather than watery). Mass motility should be a minimum of 2 out of 5. Individual progressive motility should be a minimum of 60%. With the morphology count, at least 75% normal cells should be present. Abnormalities should not exceed 25% these comprise of major and minor defects, made up of 28 different defects ranging from head, mid‐piece, and tail defects (Butler et al. 2020; Hopper 2015; Kumar et al. 2015).

The sperm swim‐up technique was used, where 10 μL of the semen sample was added to 500 μL of pre‐warmed Tris medium (37°C) (Trizma, Sigma‐Aldrich, USA). A 10 μL aliquot of the extended semen was then placed onto a pre‐warmed microscope glass slide and examined under a microscope at ×10 magnification, and sperm total motility was assessed and expressed as a percentage.

To evaluate sperm viability and morphology, an Eosin‐Nigrosin stain was used (Agarwal et al. 2016). The sperm smears were prepared on clean, pre‐warmed glass slides, allowed to air dry at room temperature (25°C), and then examined under a fluorescence microscope (Olympus, Japan). For this sample, 200 sperm cells were counted and analyzed using a DBC.6 laboratory counter (Han Lien International Corp., Taiwan). Sperm morphology was classified into two categories: normal and abnormal. Abnormal spermatozoa were further divided into primary and secondary abnormalities. Primary abnormalities occurred during spermatogenesis in the testes (head defects), secondary abnormalities during the transition from the testes to the epididymis (mid‐piece defects) (Amato and Patton 2011).

### 2.4 Vasectomy Procedure

Following standard capture procedure, the giraffe was anesthetized with 5 mg medetomidine (Kyron Labs, SA) and 5 mg thiafentanil (Thianil 10 mg/mL, Wildlife Pharmaceuticals, SA) administered intramuscularly via a Pneu‐dart projector shot from a helicopter. Once recumbent, the giraffe was blindfolded, and its legs were secured with ropes by the experienced game capture team. The effects of Thianil were immediately reversed with 2 mL of Trexonil (Naltrexone 100 mg/mL, Wildlife Pharmaceuticals, SA) administered intravenously (Deacon et al. 2022).

**Figure 1:**
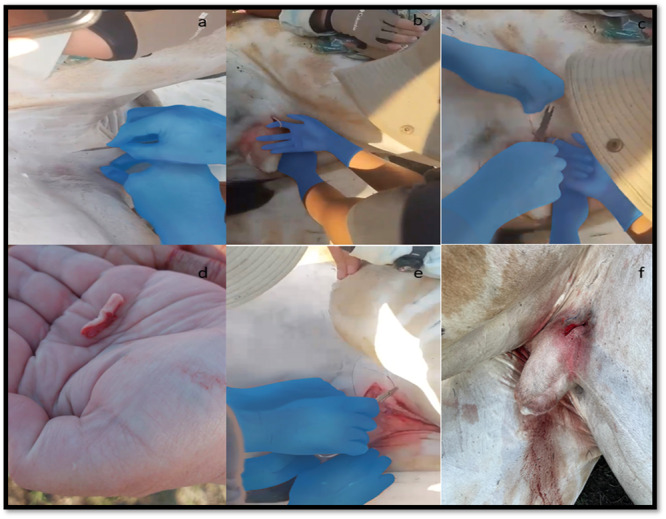
The surgical procedure followed for the vasectomy of an adult, male giraffe (*Giraffa camelopardalis giraffa*) during 2024 from a private game reserve in the Free State, South Africa. The figure illustrates (a) skin incision; (b) extrusion of vas deferens; (c) ligating vas deferens and removing it; (d) vas deferens removal; (e) closure of wound; (f) surgical wound postoperative.

The vasectomy was performed under local anaesthesia with 20 mL Lignocaine (Centaur Pharmaceuticals) injected subcutaneously in the surgically prepared area and into the spermatic cord, whereafter a skin incision was made (Figure 1). The animal was positioned in right lateral recumbency for the prevention of bloating. A 5–7 cm skin incision was made at the level of the prepuce in an anterolateral direction over the spermatic cord. The subcutaneous tissue and dartos muscle were bluntly dissected from the underlying spermatic facia (Armstrong 2023). This allowed the surgeon more room to lift the spermatic cord through the skin and fixate it with a clamp. The spermatic cord was then incised longitudinally to visualise the ductus deferens (Al Haideri et al. 2019; Althouse and Evans 1997). The ductus was isolated by dissecting the ductus away from the deoductus and pampiniform plexus. Care had to be taken when handling the pampiniform plexus, as damage to the plexus could lead to extensive bleeding.

The isolated ductus was clamped on both the testicular and the prostatic side using artery clamps, about 2 cm apart bilaterally, and a piece of 1 cm was removed. A section of the ductus was removed using surgical scissors, and the artery forceps were left in place on both sides of the remaining ductus. The resected section of the ductus deferens was placed in formalin solution and kept for histopathological confirmation. Each end of the ductus deferens was then ligated using Catgut #4 (SMI) (D'Cunha et al. 2022). The subcutis and dermis were closed together using an inverted mattress suture made of monofilament nylon. Samples of the vas deferens were preserved in 10% neutral buffered formalin for histopathological confirmation (Norris et al. 2024) (Figure 4). After the vasectomy was completed, the focus animal was treated as per standard veterinary protocol and animal research ethics approval.

### 2.5 Histology Procedure

The tissue was processed and stained using routine histological techniques (Feldman and Wolfe 2014). Briefly, the tissue was processed overnight in a Tissue‐Tek VIP 6 Tissue Processor (Sakura Finetek Inc., USA, Torrance, CA). The tissue was then manually embedded in wax blocks. Four micrometer thin sections were cut, floated on a water bath, and picked up on glass slides. The slides were then stained with hematoxylin and eosin using a Tissue‐Tek Prisma Plus Automated Slide Stainer and Tissue‐Tek Film Automated Coverslipper (Sakura Finetek Inc., USA, Torrance, CA) (Figure 4).

### 2.6 Recapture Procedure for Post‐Surgery Semen Evaluation

Five weeks post‐vasectomy, the giraffe was immobilized a third time using 17 mg of thiafentanil (Thianil 10 mg/mL, Wildlife Pharmaceuticals, SA). Electro stimulation was used again to induce ejaculation to determine the success of the procedure and to confirm vasectomy. The ejaculation sample was analyzed in a controlled laboratory setting to ensure accurate results (Absolute Genetics 2025). The lack of spermatozoa in the ejaculate confirmed the success of the procedure (Figure 5).

### 2.7 Evaluation of Frozen Ejaculation and Semen

The evaluation of frozen semen for quality control involved a standardized procedure using specified equipment, consumables, and methods to ensure accurate and consistent results (RAMSEM 2025). A phase contrast microscope equipped with a heated stage set at 38.0°C was used for semen assessment. Heated water baths were maintained at 35.5°C. A 10 µL pipette was employed for precise handling of semen samples. Consumables included pipette tips, microscope slides, and coverslips heated to 38.0°C, 15 mL glass tubes, a 100 mL glass bottle, and a plastic container filled with water for waste collection. Additional items include a semen freezing evaluation form, a pen, and 10% formalin solution for morphological analysis.

The evaluation begins with filling and preheating the water baths and the microscope's heat plates to their designated temperatures. Glass tubes for each semen straw, as per the freezing list, were pre‐warmed in the appropriate water baths. Microscope slides and coverslips were placed on the heat plate for warming. Once all equipment reached the set temperatures, the semen was thawed in batches according to the freezing list. During thawing, the time was recorded, and the selected batch of straws was removed from the nitrogen flask, excess nitrogen was shaken off, and the straws were placed into the 38°C water bath within 3 s. Straws were agitated gently in the water bath to prevent clumping and thawed for 40 s for mini straws or 60 s for medium straws. The thawed straws were transferred to a glass bottle, then removed in groups of 2–4, dried with a paper towel, and both ends were cut to drain the semen into pre‐warmed tubes.

The initial evaluation (0 h) involved preparing droplets of semen from the tubes on microscope slides. For rams, 4 µL droplets are used; for bovine bulls, 5 µL droplets. The slides were examined under the microscope at 10× magnification (PH1) to assess motility, movement, and morphological abnormalities. Observations were recorded on the evaluation form, including abbreviations for abnormalities, as then listed in a table. Sperm morphology was assessed either before freezing, during the 0‐h evaluation, or during subsequent stress tests. A 5 µL droplet of semen was mixed with 2 µL of formalin and covered with a coverslip. Under 40× magnification (PH2), sperm cells were examined to count primary and secondary abnormalities. A total of 100 or 200 cells were counted to determine the percentage of normal cells, and the results are documented.

A stress test evaluation was performed after 2 or 4 h, depending on the species (2 h for bovine). Similar to the 0‐h evaluation, motility, movement, and abnormalities were recorded. The results of the stress test, alongside the 0‐h evaluation data, were used to determine whether the batch meets quality control standards based on the parameters outlined. After evaluation, all used slides and tubes were discarded into the designated container and sent for cleaning and sterilization. These steps ensured thorough quality control and the reliable assessment of frozen semen for reproductive purposes.

### 2.8 Evaluation of Testosterone Levels

The evaluation of the testosterone levels was conducted as per standard and validated methods described in previous work (Quanson et al. 2016). Results on these findings are only briefly mentioned in the current study, as it forms part of future publications, which are currently in development.

## 3 Results and Discussion

### 3.1 Pre‐Surgery Semen Evaluation

**Figure 2:**
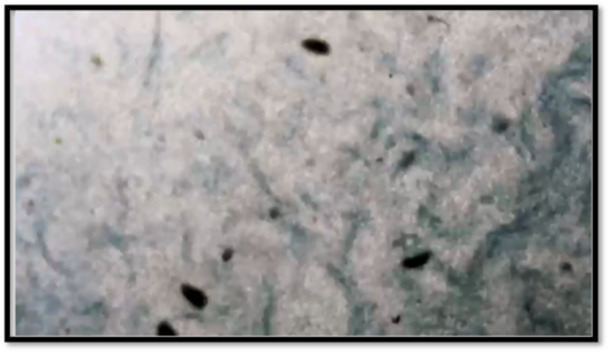
Semen examination performed prior to the vasectomy surgery of an adult, male giraffe (*Giraffa camelopardalis giraffa*) during 2024 from a private game reserve in the Free State, South Africa, The microscopy illustrates the marbling effect of semen movement (sperm agglutination).

The pre‐surgery semen analysis confirmed that the giraffe was fertile, with 59% normal sperm (Absolute Genetics 2025) (Table 1). This result indicated that the selected giraffe was an ideal candidate for a vasectomy procedure to be performed (Figure 2).

**Table 1:** A pre‐vasectomy semen evaluation report from an adult, male giraffe (*Giraffa camelopardalis giraffa*) during 2024 from a private game reserve in the Free State, South Africa (Absolute Genetics 2025).

Bull identification	Owner and collection date	% Primary abnormal	% Secondary abnormal	% Normal sperm	Remarks
Bull 4 (White) “Willem”	*Save the Giraffes*; May 20, 2024	18%	23%	59%	P: Acrosome, tails, head defects, shape heads, nuclear vacuoles S: Coiled tails, bent midpiece, bent tails.

### 3.2 Surgery Outcome

**Figure 3:**
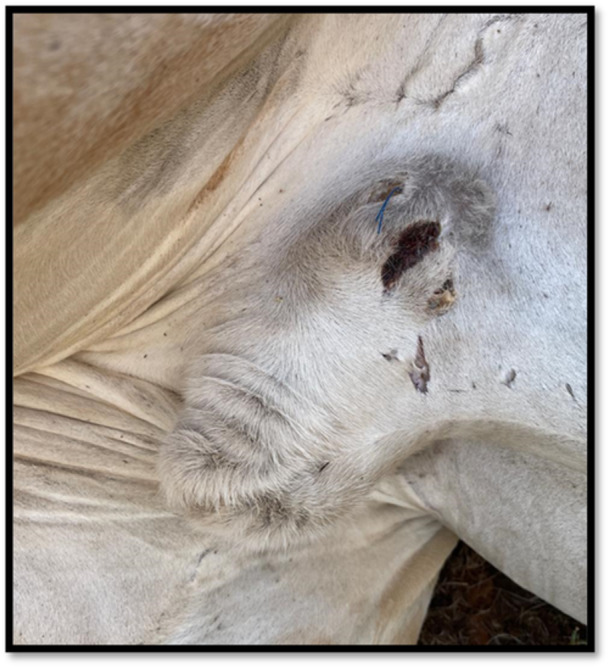
The surgical incision site, five weeks after the vasectomy procedure performed on an adult, male giraffe (*Giraffa camelopardalis giraffa*) at a private game reserve in the Free State, South Africa.

The vasectomy procedure was successful, with no postoperative complications such as incisional dehiscence or sperm granulomas observed. The surgical wound healed without obvious complications (Figure 3).

### 3.3 Histology Results

**Figure 4:**
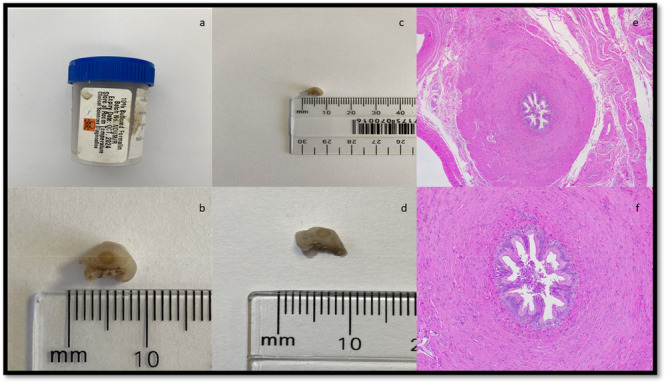
The collection of specimens from an adult, male giraffe (*Giraffa camelopardalis giraffa*) (Free State, South Africa, 2024) during a vasectomy surgical procedure for histopathological examination, illustrating the: (a) vas deferens in a formalin bottle; (b) vas deferens left side; (c) vas deferens left side; (d) vas deferens right side; (e) histology of left vas deferens; (f) histology of right vas deferens (Agarwal et al. 2016).

Histopathological examination confirmed the presence of vas deferens tissue, validating the success of the vasectomy (Figure 4). Anatomical principles outlined in a previous study centered on the cross‐sectional analysis of the vas deferens and its associated blood supply in humans (Narasimman et al. 2024). The importance of preserving the vasculature during the procedure to avoid complications such as ischemia and maintain normal hormonal function is also underscored (Narasimman et al. 2024) and provides valuable insights applicable across species, including the giraffe.

**Figure 5:**
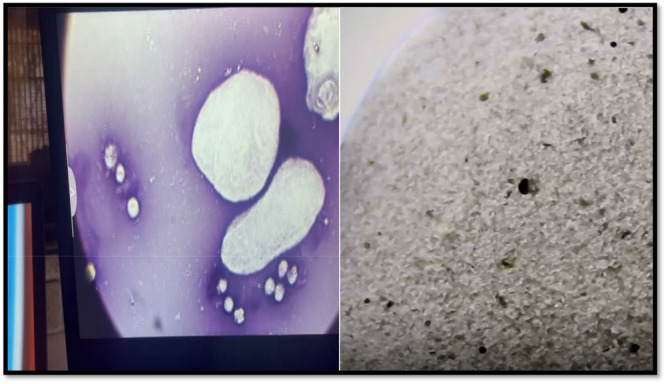
The electroejaculate sample from an adult, male giraffe (*Giraffa camelopardalis giraffa*) (Free State, South Africa, 2024) 5 weeks after the performance of vasectomy surgical procedure, indicating the absence of spermatozoa. The left sample was stained with Eosin‐Nigrosin for histopathological examination (Agarwal et al. 2016).

### 3.4 Post‐Vasectomy Semen Evaluation

The post‐vasectomy semen analysis showed no presence of spermatozoa, confirming the giraffe's infertility and the success of the surgical procedure (Narasimman et al. 2024).

### 3.5 Testosterone Levels

The pre‐ and post‐vasectomy testosterone levels of the focus animal (2.97 nmol/L and 0.86 nmol/L, respectively) (Table 2) were within the range of testosterone levels determined from other non‐vasectomised giraffe males sampled during the same study period (Table 3), indicating that the vasectomy did not adversely affect the giraffe's hormonal balance or mating behavior (Seeber et al. 2013). The pre‐ and post‐vasectomy testosterone level of the focus animal was, however, lower than the median value (8.53 nmol/L) calculated but further statistical considerations did not form part of the scope of this particular study (Table 3).

**Table 2:** Testosterone levels were determined pre‐and post the vasectomy of an adult, male giraffe (*Giraffa camelopardalis giraffa*) located on a private game reserve in the Free State, South Africa during 2024.

	Animal identification	Testosterone (nmol/L)
Pre‐vasectomy	Bull 4 (White) “Willem”	2.97
Post‐vasectomy		0.86

**Table 3:** Testosterone levels of other non‐vasectomised giraffe males (age range 6–15 years) sampled during the study period (2023–2024) located in the Free State, South Africa.

Animal identification	Testosterone (nmol/L)
M1	3.22
M2	36.48
M3	39.19
M1a	18.94
M2a	1.76
M3a	0.16
M4a	16.24
M5a	64.01
M6a	6.15
M7a	0.50
Sample 8 OOM KOBUS	8.53
Sample 9 RAUBEX BUL	64.88
Sample 12 BULL 2	8.09
Sample 14 OOM KOBUS BULL	7.95
Sample 37 BUL 1	3.18
Sample 41 BUL 2	15.72
Sample 1 DBb	13.15
Median	8.53
Standard deviation	20.14

### 3.6 Male Behavior Post‐Vasectomy

**Figure 6:**
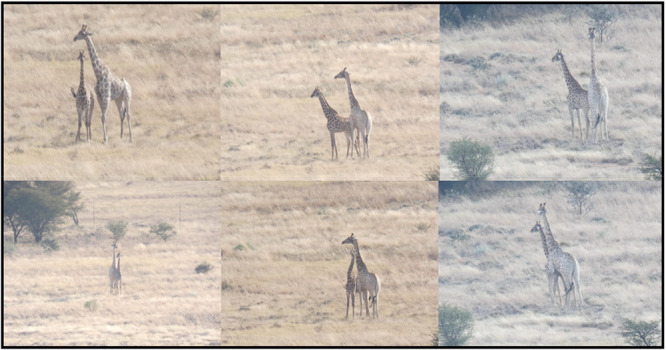
The vasectomised adult, male giraffe (*Giraffa camelopardalis giraffa*) (Free State, South Africa, 2024) showed clear guarding behavior towards a female, which was treated with oestrus synchronisation hormones.

The vasectomised adult, male giraffe showed clear guarding behavior towards a female, treated with oestrus synchronisation hormones (Figure 6), which is typical behavior displayed by male giraffes when a female is in heat (Bercovitch et al. 2006; Deacon et al. 2024; Mitchell 2021). The performed surgical procedure was therefore successful in establishing a teaser male (Morgan and Dawson 2008) to be used in future giraffe reproduction studies.

## 4 Conclusion

The successful vasectomy performed on a Southern African male giraffe marks a significant advancement in the application of vasectomy for wildlife reproductive management. This procedure effectively rendered the giraffe infertile while preserving its normal testosterone levels and sexual behavior. The study demonstrates the potential of using vasectomized giraffes in conservation strategies, such as enhancing breeding programs by facilitating more precise detection of oestrus in females, a challenge often encountered in captive and semi‐captive populations.

The study underscores the importance of meticulous surgical technique in achieving dual objectives such as sterility and hormonal normalcy. This study also provides insights into broader wildlife conservation, demonstrating that vasectomy can be a practical and effective tool for managing reproductive dynamics in non‐human species. Ultimately these findings contribute to a growing body of evidence supporting the use of vasectomy in a wide range of contexts, offering pathways for improved outcomes in both clinical and ecological applications.

## Ethics Statement

All procedures used in this study were approved by the UFS Animal Research Ethics Committee: Number UFS‐AED2023/0082/5.

## Conflicts of Interest

The authors declare no conflicts of interest.

## Data Availability

The data that support the findings of this study are available from the corresponding author upon reasonable request.
